# Adaptation of the Quiet Quitting Scale into Turkish: A methodological study

**DOI:** 10.1371/journal.pone.0357582

**Published:** 2026-10-12

**Authors:** Nuray Kurt, Gülcan Bahcecioglu Turan, Mizgin Demir

**Affiliations:** 1 Department of Midwifery, Faculty of Health Sciences, Fırat University, Elazığ, Türkiye; 2 Department of Nursing, Faculty of Health Sciences, Fırat University, Elazığ, Türkiye; College of Nursing, Qassim University, SAUDI ARABIA

## Abstract

Quiet quitting has emerged as a contemporary work behavior associated with reduced organizational engagement and increased work-related stress, particularly among healthcare professionals. This study aimed to adapt the Quiet Quitting Scale into Turkish and evaluate its validity and reliability among healthcare workers. This methodological study was conducted online between February and April 2025. The sample included 298 healthcare professionals currently working in Turkey. Data were collected using the “Personal Information Form” and the “Quiet Quitting Scale.” Content validity analysis showed item-level I-CVI values ranging from 0.83 to 1.00, with a total scale S-CVI of 0.97. Factor loadings for individual items ranged from 0.510 to 0.890. Confirmatory factor analysis (CFA) confirmed that the proposed factor structure fitted the data well, with model fit indices as follows: χ² = 64.31; df = 21; p < 0.05; χ²/df = 3.06; RMSEA = 0.083; CFI = 0.97; SRMR = 0.052; TLI = 0.95; RMR = 0.069; AIC = 112.31. These findings provide strong support for the structural validity of the scale. The Turkish version of the Quiet Quitting Scale demonstrates both validity and reliability, making it a suitable tool for evaluating quiet quitting tendencies among healthcare workers.

## Introduction

In recent years, with the changing expectations regarding work life, the differing meanings employees assign to their jobs, and the emphasis on work-life balance, new work behaviors have emerged. One of these behaviors, “Quiet Quitting,” is defined as employees fulfilling only the responsibilities outlined in their job descriptions without completely leaving their jobs and consciously avoiding going beyond their work roles [1]. Quiet quitting is not a genuine resignation behavior; rather, it refers to a silent withdrawal process where employees prefer to perform at a minimum level at work, establish boundaries to protect their work-life balance, and avoid taking on additional responsibilities [2].

Fundamentally, quiet quitting is considered a coping and protection strategy developed by employees in response to the intense work culture, increasing workload, and experiences of burnout. This approach reflects employees prioritizing their individual well-being over corporate expectations and developing an awareness that life is not solely about work [2]. While traditional turnover behavior refers to employees physically leaving the organization, quiet quitting is characterized by a decrease in organizational commitment and voluntary contributions, even though employees continue to stay at work [3]. This situation can negatively affect employee performance and organizational efficiency over time [4].

The healthcare sector is one of the fields where many occupational stressors coexist, such as heavy workloads, long working hours, shift work systems, staff shortages, burnout, risk of violence and mobbing, low wages, the possibility of work accidents, and the inability to establish a sustainable work-life balance [3,5,6]. These unfavorable working conditions can affect healthcare workers’ attitudes toward their jobs, leading to decreased job satisfaction, weakened organizational commitment, and increased intention to leave their jobs [7–9]. Especially during the COVID-19 pandemic, an increase in burnout, anxiety, and depression levels among healthcare workers was observed, along with a decrease in job commitment, and it was reported that silent resignation behaviors became more visible [10].

Healthcare services, due to their direct dependence on employee engagement and performance, can be more affected by quiet quitting behavior compared to other sectors [6]. The widespread silent resignation behavior among healthcare workers can lead to a decrease in work efficiency, a decline in the quality of care, a risk to patient safety, and an increase in healthcare service costs [7–9,11]. Therefore, it is of great importance for healthcare institutions to identify this change in employee attitudes at an early stage and to develop appropriate human resources strategies [12].

The scientific evaluation of quiet quitting behavior requires the use of valid and reliable measurement tools. The Quiet Quitting Scale (QQS), developed by Galanis and colleagues (2023), is one of the contemporary and theoretically grounded measurement tools that assess employees’ tendencies to limit their work roles and changes in their levels of organizational engagement [11]. However, cultural and linguistic adaptation studies are necessary for the scales to be used in different societies. In Turkey, especially among healthcare workers, a valid and reliable measurement tool for assessing the behavior of silent resignation has not been found. This situation limits the objective determination of healthcare workers’ tendencies toward silent resignation and the development of evidence-based intervention strategies.

THIS STUDY FOCUSED ON PRODUCING A TURKISH VERSION OF THE QQS [11] AND EVALUATING ITS MEASUREMENT ACCURACY AMONG HEALTHCARE PROFESSIONALS.

Therefore, the aim of this study was to translate and culturally adapt the Quiet Quitting Scale (QQS) into Turkish and to evaluate its psychometric properties, including validity and reliability, among healthcare professionals in Türkiye.

## Materials and methods

### Design

The research is methodological research.

### Participants and sample

The data for the research were collected online between February 2025 and April 2025. Participants were recruited using a convenience sampling strategy combined with snowball sampling through various online platforms and professional networks. A form prepared via Google Forms was used as the data collection tool and was distributed to healthcare workers actively serving across Turkey. Before the study, participants received detailed information about the research and provided their voluntary informed consent online. The inclusion criteria were being actively employed in a healthcare institution in Turkey and completing all items on the survey form. To maintain data quality, all responses were reviewed before analysis, and questionnaires completed in less than 60 seconds were excluded because this duration was considered insufficient to read and respond carefully to all questionnaire items, indicating potential random responding. In the initial phase of the study, 880 people were contacted. Of these, 222 were excluded for not meeting the eligibility criteria, and 360 declined to provide consent. As a result, the final validation sample consisted of 298 participants.

Adequate sample sizes for assessing a scale’s validity and reliability are typically several times larger than the number of items it contains [13]. Since the original form of the “Silent Quitting Scale” consists of 9 items, a sample size of at least 45–90 people is considered sufficient. In this context, it has been determined that the sample size of 298 individuals is statistically quite sufficient in terms of the significance and generalizability of the analyzes.

### Data collection tools

The data were collected using the “Personal Information Form” and “The Quiet Quitting Scale.”

**Personal Information Form:** In the form prepared for the participants, there are questions aimed at collecting demographic and profession-related information such as age range, gender distribution, education level, occupation, and years of work.

**The Quiet Quitting Scale (QQS):** The QQS is a 5-point Likert-type scale that possesses robust psychometric properties and consists of 9 items and 3 factors. The scale is employed to quantify employee reticence. Items 7, 8, and 9 are evaluated in reverse order. Factors: Detachment (items 1, 2, 3, 4); lack of initiative (items 5, 6, 7); and lack of motivation (items 8, 9). Each score is assigned a value between one and five. A quieter ending is associated with higher scores. 2.06 is the suggested cutoff point for the total score. Employees who have a total score of ≥2.06 are regarded as quitting quietly, while those who have a total score of <2.06 are not deemed to be quitting quietly [11].

### The process of adapting the scale to Turkish

The scale was adapted into Turkish through a three-step process, which involved assessing language equivalence as well as conducting validity and reliability analyses. The translation and cross-cultural adaptation process was conducted in accordance with the guidelines proposed by Beaton et al. (2000) and the COSMIN recommendations for the cross-cultural adaptation of patient-reported outcome measures [14].

**In the First Stage;** Two English language specialists translated the nine items from the scale’s original form into Turkish. Any discrepancies between the two forward translations were resolved through discussion and consensus among the research team. Careful consideration was given to the preservation of conceptual and cultural appropriateness throughout the translation process. In order to assess the content validity of the draft form, a six-member expert panel comprised of psychologists, midwives, nurses, and linguists was sought. The expert evaluations determined that the content validity index (CVI) of all items was greater than 0.80. Consequently, no items were eliminated from the scale. The form was retranslated into English by two distinct language experts (back-translation) after the linguistic corrections were implemented in accordance with expert recommendations. The meaning was confirmed to be consistent with the original text, and the pilot application phase was subsequently initiated.

**In the second phase,** A preliminary application was conducted on a pilot group of 34 individuals in the second phase to evaluate the comprehensibility of the items. Within the scope of reliability analysis, it was adopted as a basic criterion that item-total correlations should be above the threshold of 0.30 and the Cronbach’s alpha coefficient should be above the threshold of 0.70 [13,15]. It was determined that the values in the pilot application were above these thresholds, and the main application was proceeded with. The participants included in the pilot study were not included in the main psychometric validation sample.

**In the third phase,** structural validity analyses were conducted using data obtained from 298 participants. As a result of the CFA, the fit indices were compared with the criteria recommended in the literature [16,17], and the structural validity of the scale was confirmed. To determine the scale’s temporal stability, a test–retest analysis was conducted with a 15-day interval using a randomly selected subgroup of 34 participants from the main validation sample (N = 298), in accordance with the recommended 15–30-day interval [13]. The participants included in the pilot study were not included in the main validation sample. The results demonstrated that the Turkish version of the scale is a consistent and reliable measurement tool over time.

### Statistical analysis

The data were captured using Google Forms and subsequently transferred to Excel for analysis using SPSS 27 and LISREL programs. Cronbach’s alpha values and item-total correlations were assessed. The data’s suitability for analysis was assessed using the KMO coefficient and Bartlett’s test of sphericity. The sample was deemed adequate due to the significance of the Bartlett test and the KMO value exceeding 0.60 [13].

Confirmatory factor analysis (CFA) was conducted to evaluate construct validity without performing exploratory factor analysis (EFA), as the aim of this study was to test the fit of the original, theoretically established factor structure rather than to explore a new latent structure. When the original factor structure has been previously established, CFA is recommended to verify whether the hypothesized measurement model can be replicated in a different language and cultural context rather than conducting a new exploratory analysis [18]. Accordingly, the use of CFA without prior EFA is consistent with the COSMIN recommendations for evaluating the structural validity of existing instruments in cross-cultural adaptation studies [19].Similar methodological approaches have been adopted in previous validation studies of established measurement instruments [20–24].

The fit indices CMIN/DF, RMSEA, CFI, TLI, SRMR, and RMR were employed to evaluate construct validity in the LISREL program [16,17,25]. The reliability of the scale was evaluated using Cronbach’s alpha and McDonald’s Omega, with coefficients above 0.70 indicating acceptable reliability [13]. Furthermore, the test-retest method has been implemented to evaluate its temporal stability.

### Ethics of the research

Approval for the study was granted by the university’s Ethics Committee (Ethics Committee Approval No: 05.02.2025–31259). After obtaining ethical approval, no further institutional authorization was deemed necessary because the data were collected online from individuals not affiliated with a specific institution. Permission to translate the QQS into Turkish was obtained from its original author. The research adhered to the ethical principles outlined in the Helsinki Declaration, and all participants were informed about the study’s purpose and gave their consent online.

## Finding

It was noted that 29.2% of the participants were in the 26–30 age range, 82.9% were women, 60.7% were bachelor’s degree holders, 35.2% were nurses by profession, and 56% had 0–10 years of work experience (Table 1).

**Table 1:** Descriptive characteristics of participants.

Characteristics	Number (n = 298)	%
**Age**		
18-25	43	14.4
26-30	87	29.2
31-35	83	27.9
36-40	36	12.1
41 ve üzeri	49	16.4
**Gender**		
Female	147	82.9
Male	51	17.1
**Education status**		
Vocational high school	1	3.4
Associate degree	28	9.4
Bachelor’s degree	161	60.7
Postgraduate degree	79	26.5
**Profession**		
Nurse	105	35.2
Midwife	71	23.8
Doctor	35	11.7
Dietitian	21	7.0
Physiotherapist	23	7.7
Pharmacist	3	1.0
Other	40	13.4
**Years of service**		
0-10 years	167	56.0
10-20 years	97	29.2
20-30 years	37	12.4
30 years and above	7	2.3

Other = Health technician.

### Findings related to validity

#### Content validity.

Based on expert evaluations, the item-level I-CVI scores varied between 0.83 and 1.00, and the overall scale-level S-CVI was determined to be 0.97

#### Structural validity.

Prior to examining structural validity, the KMO measure and Bartlett’s Test of Sphericity were used to assess sample adequacy and determine whether the data were appropriate for factor analysis. The KMO value of 0.823 indicated sufficient sampling, while Bartlett’s test showed significant results (χ² = 883.072; p < 0.001). To confirm the three-factor structure of the nine-item scale, CFA was conducted directly without performing an EFA. The CFA results demonstrated factor loadings between 0.510 and 0.890, supporting the appropriateness of the three-factor model (Table 2).

**Table 2:** Mean, ıtem correlation coefficient, and DFA factor load results.

Scale Items	Mean±SD	Corrected Item-total Correlations	Factor Loading Value		
			F1	F2	F3
Item 1	2.58 ± 1.22	0.627	0.740		
Item 2	2.89 ± 1.26	0.512	0.630		
Item 3	2.31 ± 1.09	0.585	0.630		
Item 4	2.00 ± 1.14	0.479	0.510		
Item 5	2.40 ± 1.18	0.582		0.700	
Item 6	2.75 ± 1.29	0.582		0.780	
Item7	2.62 ± 1.13	0.585		0.740	
Item8	2.56 ± 1.09	0.385			0.660
Item9	3.00 ± 1.19	0.506			0.890

### Confirmatory factor analysis

The CFA yielded the following fit indices: χ² = 64.31, df = 21 (p < 0.05), χ²/df = 3.06, RMSEA = 0.083, CFI = 0.97, SRMR = 0.052, TLI = 0.95, RMR = 0.069, and AIC = 112.31 (Table 3). Following examination of the modification indices, correlated residuals were specified between Items 3 and 4, Items 4 and 5, and Items 6 and 7 because these item pairs belong to the same latent factor and assess conceptually related aspects of quiet quitting. The standardized residual covariance estimates shown in Fig 1 were 0.19, 0.20, and −0.18, respectively. These theoretically justified modifications improved the overall model fit while preserving the original three-factor structure of the scale. The corresponding standardized path diagram is presented in Fig 1.

**Table 3:** Confirmatory factor analysis results.

Fit criteria	Found	Appropriate	Acceptable	Results
*x*^2^/df (**CMIN/DF)**	3.06	<2	<5	Acceptable adaptation
RMSEA	0.083	<0.05	<0.08	Acceptable adaptation
CFI	0.97	>0.95	>0.90	Perfect harmony
SRMR	0.052	<0.05	<0.08	Acceptable adaptation
RMR	0.069	<0.05	<0.08	Acceptable adaptation
TLI	0.95	>0.95	>0.90	Acceptable adaptation
AIC	112.31			

AIC: Akaike Information Criteration.

**Fig 1:**
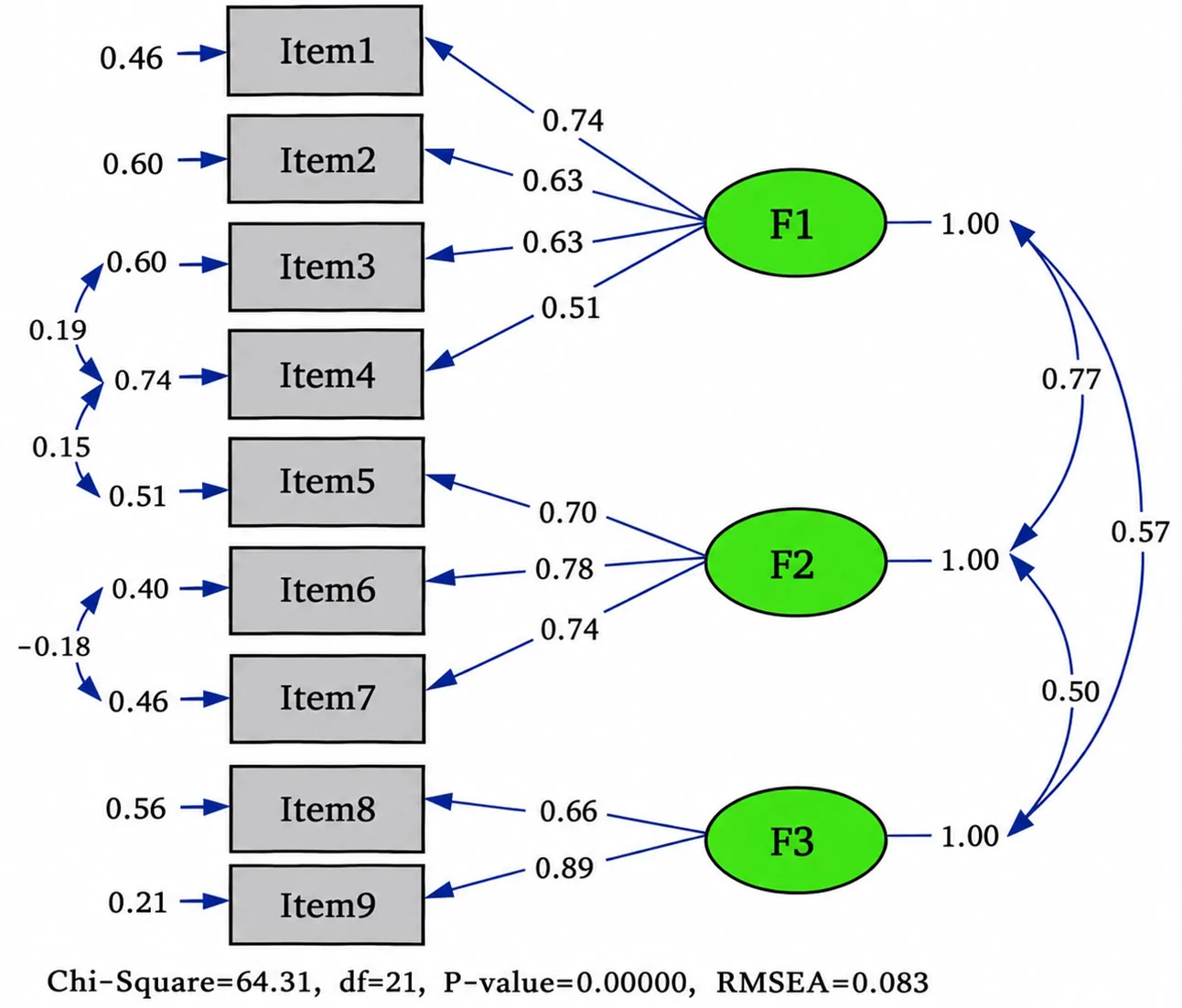
PATH diagram regarding the factor structure of the scale.

Convergent validity was assessed using the Average Variance Extracted (AVE) and Composite Reliability (CR). The AVE values ranged from 0.401 to 0.614, while the CR values ranged from 0.724 to 0.784, indicating acceptable convergent validity overall. Discriminant validity was further evaluated using the Fornell–Larcker criterion. The square root of the AVE for each construct is presented on the diagonal of Table 4, whereas the off-diagonal elements represent the latent factor correlations. The criterion was fully satisfied for the third factor, whereas the relatively high correlation between the first and second factors indicated a close conceptual relationship between these dimensions.

**Table 4:** Inter-factor correlations, Fornell–Larcker criterion, and reliability indices.

Scale and sub-dimensions	F1	F2	F3	AVE	CR	α	Ω
**F1**	**0.633**			0.401	0.724	0.744	0.736
**F2**	0.770	**0.741**		0.549	0.784	0.738	0.747
**F3**	0.570	0.500	**0.784**	0.614	0.757	0.738	–
QQS Total	–	–	–			0.832	0.825

Abbreviations: F = factor; F1 = Detachment; F2 = Lack of initiative; F3 = Lack of motivation; AVE = Average Variance Extracted; CR = Composite Reliability; α = Cronbach’s alpha; Ω = McDonald’s omega. Diagonal values (bold) represent the square root of the AVE (Fornell–Larcker criterion); off-diagonal values represent latent factor correlations obtained from the CFA model.

### Findings on reliability

Reliability analysis showed that the scale’s sub-dimensions had Cronbach’s alpha values between 0.738 and 0.744, while the total scale achieved 0.832. Omega coefficients were similarly in the range of 0.736 to 0.825 (Table 4), and item-total correlations ranged from 0.385 to 0.627 (Table 2).

Temporal stability was assessed via a test-retest with 34 participants over 15 days. The correlations between the two time points were high, ranging from 0.863 to 0.916, all statistically significant (p < 0.01). No significant differences were found between the first and second measurements (p > 0.05). Intraclass correlation coefficients for test-retest reliability ranged from 0.856 to 0.906 (Table 5).

**Table 5:** Test-retest results and score averages (n = 34).

Scale	Scale Score Means		Analysis Results				ICC
	First Implementation X ± SD	Second Implementation X ± SD	r	p	t	p	
F1	2.30 ± 0.85	2.20 ± 0.67	0.883**	0.000	1.468	0.151	0.924
F2	2.33 ± 0.80	2.36 ± 0.75	0.863**	0.000	−0.415	0.681	0.925
F3	1.47 ± 0.49	1.52 ± 0.50	0.916**	0.000	−1.676	0.103	0.956
QQS Total	2.13 ± 0.57	2.10 ± 0.45	0.850**	0.000	0.442	0.661	0.906

**Correlation is significant at the 0.01 level (2-tailed); r = Pearson Correlation Coefficient; t: t = Paired sample t test; F = factor; F1 = Detachment; F2 = Lack of initative; F3 = Lack of motivation.

## Discussion

Healthcare workers can experience significant levels of stress and burnout due to reasons such as heavy workloads, long working hours, and high responsibilities. It has been reported that this situation increases job dissatisfaction, triggering the intention to leave the job and the increasingly discussed behavior of “quiet quitting” in recent years [11]. In this regard, assessing quiet quitting requires tools that are both reliable and valid. This study aimed to translate the scale designed to measure silent resignation into Turkish and to evaluate its validity and reliability among healthcare professionals.

Content validity is an important stage in scale adaptation studies, as it determines whether the items adequately represent the structure to be measured [26,27]. In this study, the item-level I-CVI values ranged between 0.83 and 1.00, and the scale-level S-CVI value was found to be 0.97, indicating that the Turkish form has strong content validity [27]. In the Chinese adaptation study of the scale, the S-CVI value was reported as 0.989, indicating that the obtained findings are consistent with the literature [28]. These values, obtained above the acceptable limits proposed in the literature, support the successful completion of the translation and cultural adaptation process [26,27].

If the scale has been previously validated and the factor structure is known in advance, it is recommended to apply CFA directly instead of EFA [29]. Before the CFA, the KMO and Bartlett’s sphericity tests were applied. The KMO value obtained in this study is 0.823, and the result of Bartlett’s test of sphericity (χ² = 883.072; p < 0.001) indicates that the data is suitable for factor analysis. In the original study, the KMO value was reported as 0.845 and the Bartlett test result as p < 0.001, with both studies presenting similar findings in terms of high sample adequacy and suitability for factor analysis [11,15].

The CFA results, showing that the factor loadings range between 0.510 and 0.890, indicate that all items represent the relevant factors at a strong and significant level; therefore, there was no need to remove any items [15]. Fit indices (CMIN/DF = 3.06, RMSEA = 0.083, CFI = 0.97, SRMR = 0.052, TLI = 0.95, RMR = 0.069) fall within the acceptable and good fit ranges recommended in the literature, supporting the multidimensional structure of the model [30–32]. In addition, a small number of correlated residuals were specified between Items 3–4, Items 4–5, and Items 6–7 based on modification indices and theoretical justification. Because these item pairs belong to the same latent factor and reflect conceptually related aspects of quiet quitting, allowing their residuals to correlate was considered appropriate. These modifications improved the overall model fit while preserving the original three-factor structure and the conceptual integrity of the scale. The fit values reported in the original study (CMIN/DF = 3.184, CFI = 0.957, TLI = .939, RMSEA = 0.069) [11] showing similarity indicate that the structural validity of the Turkish version of the scale has been preserved. The preservation of the original three-factor structure may also reflect similarities between the Turkish and Greek healthcare contexts. In both countries, healthcare professionals work under demanding working conditions characterized by heavy workloads, workforce shortages, high emotional demands, and increasing concerns regarding work–life balance. These shared occupational challenges may contribute to the comparable manifestation of quiet quitting across the two cultural settings and support the cross-cultural applicability of the construct. Convergent validity was generally supported by the composite reliability values, although the AVE value for F1 was below the recommended threshold of 0.50. Nevertheless, the CR value for F1 exceeded the recommended criterion of 0.70, indicating acceptable construct reliability despite the relatively low AVE. According to the Fornell–Larcker criterion, the latent correlation between the Detachment and Lack of Initiative factors exceeded the square roots of their AVE values, suggesting that these two dimensions are conceptually closely related and may share substantial variance [33]. Nevertheless, the factors were retained as separate dimensions because they represent theoretically distinct aspects of quiet quitting and the original three-factor structure was preserved. Future studies should further examine the distinction between these two factors in different occupational and cultural samples.

In this study, it was determined that the correlation coefficients between the sub-dimensions ranged from 0.366 to 0.620. The correlations being below 0.90 indicate that the sub-dimensions are conceptually distinct from each other and that discriminant validity has been achieved [34]. In the original study, the correlation coefficients were reported to be between 0.51 and 0.81 [11], which is consistent with the current findings and indicates that the relationships between the dimensions of the scale are at a similar level. This situation supports the preservation of the structural integrity of the Turkish form.

Reliability was assessed by examining the scale’s internal consistency through Cronbach’s α and McDonald’s omega coefficients. In this study, the Cronbach’s α values for the subscales were found to be between 0.738 and 0.744, and for the overall scale, it was found to be 0.832, indicating that the scale has a good level of internal consistency [35]. In the original study, the Cronbach’s α values of the sub-dimensions were reported to be between 0.706 and 0.707 and the total scale’s Cronbach’s α value was reported as 0.803 [11], while the general Cronbach’s α value of the Chinese version of the scale was found to be 0.856. This indicates that the obtained findings are consistent with the literature. Additionally, in this study, McDonald’s Omega values for the sub-dimensions were found to be between 0.736 and 0.747 and 0.825 for the overall scale, providing further evidence supporting the scale’s reliability. In the original study, McDonald’s Omega coefficients ranged from 0.711 to 0.806 [11], whereas the Chinese adaptation reported an Omega of 0.887, suggesting the scale produces consistent results across diverse populations [28]. These findings indicate that the Turkish version is also a reliable instrument. Although Item 4 demonstrated the lowest item–total correlation (r = 0.479), this value remained well above the recommended minimum threshold of 0.30, indicating that the item contributed adequately to the overall construct. Furthermore, removing this item did not improve the internal consistency of the scale, and it was therefore retained to preserve the conceptual integrity and comparability of the Turkish version with the original instrument.

In the test-retest procedure, 34 participants completed the scale twice over a 15-day period. The resulting correlation (r = 0.850, p < 0.001) demonstrated the scale’s stability over time. Moreover, ICC values for the sub-dimensions ranged from 0.924 to 0.956, with 0.906 for the overall scale, indicating strong temporal reliability and consistent results across repeated administrations [36]. Temporal reliability in the original study was extremely high, with ICCs ranging from 0.987 to 0.992 for the subscales and 0.993 for the overall scale [11]. The Chinese adaptation reported an overall ICC of 0.851, which is largely in line with the findings of the current research [28].

### Limitations

Among the limitations of this study, the fact that the sample was limited to healthcare workers may restrict the generalizability of the findings to other professional groups or the general population. Additionally, data were collected using self-report measures, which may be subject to response bias and social desirability bias. Furthermore, because the data were collected online, healthcare workers with limited internet access may have been underrepresented. The response rate of 33.9% may also have introduced non-response bias, potentially affecting the representativeness of the sample. Finally, although the overall psychometric findings supported the three-factor structure of the scale, the Fornell–Larcker criterion was not fully satisfied for the Detachment and Lack of Initiative factors because of their relatively high latent correlation. Therefore, the discriminant validity between these two dimensions should be interpreted with caution, and future studies should further evaluate the factor structure using independent samples and additional methods of assessing discriminant validity. In addition, predictive validity was not evaluated in the present study. Future research should examine the ability of the Turkish version of the QQS to predict relevant organizational and occupational outcomes, such as turnover intention, burnout, job satisfaction, and organizational commitment.

## Conclusion

The findings obtained from this research have revealed that the Turkish version of the QQS exhibits a structure consistent with the original scale and serves as a highly valid and reliable tool for measuring the quiet quitting behaviors of healthcare workers. The results regarding the content and construct validity of the scale indicate that the identified sub-dimensions successfully represent the different aspects of the phenomenon of quiet quitting and that the scale is fully compatible with Turkish culture. Additionally, the internal consistency coefficients, McDonald’s Omega values, and data obtained from test-retest analyses have proven that the scale has both strong measurement precision and temporal stability. In line with these results, it is recommended that the Silent Resignation Scale be used as a routine assessment tool to detect employees’ work attitudes and potential loyalty issues early in healthcare institutions. In this way, corporate strategies and psychosocial support programs aimed at increasing employee productivity and motivation can be grounded in scientific evidence. Although this study was conducted specifically on healthcare workers, the scale’s strong psychometric properties indicate that it can be adapted to examine the phenomenon of quiet quitting in different professional groups and sectors in the future. Comparative studies with different sample groups will contribute to a deeper understanding of the relationship between this behavior and sectoral and cultural variables. In conclusion, this scale, which has been introduced to Turkish, serves as a practical, reliable, and scientific measurement tool for studies to be conducted in the fields of health management and organizational psychology.
